# The expression of sirtuins, superoxide dismutase, and lipid peroxidation status in peripheral blood from patients with diabetes and hypothyroidism

**DOI:** 10.1186/s12902-019-0350-y

**Published:** 2019-02-08

**Authors:** Abdullah Al-Khaldi, Samar Sultan

**Affiliations:** Faculty of Applied Medical Sciences, King Abdulaziz University, Jeddah, Saudi Arabia

**Keywords:** Sirtuins; Diabetes, Hypothyroidism, Superoxide dismutase, Oxidative stress

## Abstract

**Background:**

Sirtuin 1 (SIRT1) and sirtuin 3 (SIRT3) proteins have an important role in counteracting oxidative stress. Although diabetes and hypothyroidism (HT) are both characterized by oxidative stress, the mechanisms are not fully understood. This study investigated the effects of type 1 diabetes (T1D), type 2 diabetes (T2D), and HT on the expression levels of SIRT1, SIRT3, and manganese superoxide dismutase (SOD2).

**Methods:**

Gene expression of *SIRT1, SIRT3,* and *SOD2* was measured using real-time PCR. The protein expression of SOD2 and lipid peroxidation (thiobarbituric acid reactive substances) was measured by the TBARS Assay kit and enzyme-linked immunosorbent assay (ELISA) respectively.

**Results:**

The results showed that the SIRT1 and SIRT3 levels were lower in peripheral blood samples from patients with T1D, T2D, or HT than in healthy individuals. Interestingly, the mRNA and protein expression levels of SOD2 were higher in all three patient groups. Lipid peroxidation was higher in the patients with HT than in the healthy individuals.

**Conclusions:**

These results indicate alterations in the expression levels of sirtuins and superoxide dismutase in diabetes and HT, which may be related, at least in part, to the oxidative stress. Identifying such alterations in those patients will pave the way towards the development of drugs to enhance SIRT1 and SIRT3 expression and their activity to prevent the damaging effect of oxidative stress.

## Background

Sirtuins are NAD^+^-dependent histone/protein deacetylases consist of seven subtypes that are placed in the mitochondria (SIRT3, SIRT4, and SIRT5), nucleus (SIRT1, SIRT6, and SIRT7), and cytoplasm (SIRT2) [1]. Among them, SIRT1 and SIRT3 have the strongest deacetylase activities, whereas the other subtypes have weak or no deacetylase activity [1]. In this study, we focused on SIRT1 and SIRT3 because of their cytoprotective role as antioxidants in reducing oxidative stress.

There is growing evidence supporting the role of sirtuins in responding to oxidative stress through direct deacetylation of transcription factors that regulate antioxidant genes. It has been shown that SIRT1 attenuates oxidative stress in coronary arterial endothelial cells exposed to elevated glucose levels [2] and in rats with streptozotocin-induced diabetes [3]; therefore, SIRT1 may exert protective effects against diabetes and its complications. Downregulation of SIRT1 and increased systemic oxidative stress have been reported in lymphocytes and endothelial progenitor cells of T2D [2, 3]. Besides its role as an antioxidant, SIRT1—along with SIRT3—is the key regulator of glucose homeostasis [4], insulin secretion [5] and mitochondrial biogenesis [6]. SIRT3 modulates skeletal muscle mitochondrial metabolism and reactive oxygen species (ROS) production through insulin signaling in T1D and T2D [7]. It also stimulates the target gene of PGC-1α, modulates ROS production, and mitochondrial biogenesis [8]. It has been reported that SIRT3 triggers the activation of SOD2, the activity of which is suppressed by the deletion of the *SIRT3* gene [7]. In addition, *SIRT3* gene knockdown has been shown to increase apoptosis and oxidative stress levels in pancreatic islet-derived beta cells from patients with T2D [9].

Diabetes is a multifactorial complex metabolic disease characterized by impaired metabolism of carbohydrates, lipids, and proteins owing to defects in either insulin action or insulin secretion or both [10]. Worldwide around 171 million were affected by diabetes in 2000 and this is predicted to increase to 366 million in 2030 [11]. There are two major classes of diabetes: T1D and T2D. The main causes of T2D are resistance to the action of insulin accompanied by a deficiency in insulin secretion [12]. T1D is caused by the autoimmune destruction of pancreatic beta cells, resulting in a near-total deficiency of insulin secretion, and individuals with this type of diabetes are required to inject insulin [13, 14]. A disturbance in the balance of ROS levels and the antioxidative defense system, termed oxidative stress, has been shown to be linked to insulin resistance. An increase in ROS levels triggers the activation of stress kinases (e.g., c-Jun N-terminal kinase and protein kinase C), which causes the phosphorylation of insulin receptor-1 that in turn accelerates its degradation, leading to oxidative stress-induced insulin resistance [15]. In addition, studies have shown the beneficial effects of antioxidants in reversing insulin resistance and enhancing insulin sensitivity, as reported in patients with T2D who were treated with Vitamin C, Vitamin E, *N*-acetylcysteine, alpha-lipoic acid, and glutathione [16–20].

HT is caused by the reduced activity or production of thyroid hormone (TH), which results in a total decline of the metabolic process [21]. The THs T3 and T4 play key roles in regulating hepatic lipid, cholesterol, and glucose metabolism [22, 23]. Administration of T4 medication has been shown to activate T3, which in turn upregulates the important factors (e.g., SIRT1) that are involved in glucose homeostasis [24]. Furthermore, HT has been shown to be accompanied by oxidative stress [25, 26]. Increases in the activity of SOD and expression of SOD1 have been evidenced in the kidney of hypothyroid rats during postnatal development and maturation, as compared with the levels in the controls [27]. To the best of our knowledge, there is no study that has investigated SIRT3 levels in patients with HT. Given the roles of SIRT1 and SIRT3 in the regulation of mitochondrial ROS production, this study aimed to elucidate whether alterations on the expression levels of SIRT1, SIRT3, SOD2, and lipid peroxidation status are related to the observed oxidative stress in T1D, T2D, and HT.

## Methods

### Subjects

Blood samples were obtained from 22 patients with T2D (10 males and 12 females), 5 with T1D (3 females and 2 males), and 7 with HT (all females), as well as from 13 healthy donors (controls), who attended the diabetic center at King Fahad Armed Forces Hospital during the period from October 2017 – January 2018. The control subjects were non-smokers, did not take medication, did not have high blood pressure or a family history of diabetes. For the diabetic group, subjects with severe disease complications (e.g. myocardial infarction) and/or presenting with both diabetes and HT were excluded. Subjects with a fasting plasma glucose concentration of > 7 mmol/L and HbA1c > 6.5% were considered as diabetic according to the American Diabetes Association [28]. Diabetic group was on insulin alongside different drugs, such as generic metformin, NovoMix, Glucophage, and Lantus. For the HT group, subjects with diabetes and other serious diseases (e.g. cardiac disease) were excluded. Serum thyroid-stimulating hormone (TSH) determination is important for evaluating the function of the thyroid gland. Patients with serum TSH > 6 mIU/L were classified as having HT. The exclusion of subjects was achieved after clinical assessment and biochemical analysis. All the patients with HT were on thyroxine medication. Patient data including medications, duration of diseases, and anthropometric measurements such as weight, height, and body mass index were recorded from the medical patients’ profile. For experiments, including ELISA and TBARS, samples were obtained from a minimum of three individuals from each study group.

### Biochemical analysis

Eight milliliters of peripheral blood sample were collected in two tubes (+EDTA and plain) from participants of all four study groups, after overnight fasting. The EDTA sample was divided into equal volumes for RNA extraction and HbA1c analysis. A sample of the extracted RNA was analyzed within 6 h of collection to avoid RNA degradation. Serum from the plain tubes was separated by centrifugation at 3000 g for 15 min, and split into equal volumes for measurement of biochemical analytes, such as fasting blood glucose, and for other experiments such as ELISA and TBARS. The serum was stored at − 80 °C until use.

The fasting plasma glucose, TSH and cholesterol concentrations were measured by an automated enzymatic method (Cobas 8000 Modular Analyzer, Roche Diagnostics, Mannheim, Germany). The level of circulating HbA1c was measured by high-performance liquid chromatography (Variant II Turbo, Bio-Rad, Hercules, CA, USA). HbA1c normal values have ranged from 4.0 to 6.0% as reported previously [29].

SOD2 and TBARS serum concentrations were quantified with an ELISA system (SOD2 ELISA, Cusabio Co., Suffolk, UK) and TBARS assay kit (Cayman Chemical, Ann Arbor, MI, USA), respectively, following the manufacturers’ instructions.

### RNA extraction and real-time PCR

Total cellular RNA was isolated from the blood samples in EDTA tubes using the MagNA Pure Compact RNA Isolation Kit (Roche Diagnostics, GmbH) on the automatic isolator MagNA Pure Compact (Roche Diagnostics, GmbH). 1 μg of RNA was used to synthesize cDNA using an ImProm-II Reverse Transcription System kit (Promega, Southampton, UK), according to the manufacturer’s instruction. The sequences of *SIRT1*, *SIRT3*, *SOD2*, and beta-actin (reference gene) primers were as stated in our previously published study [30]. The mRNA levels among the samples were normalized to the corresponding results of the internal control Beta-actin mRNA. A QuantiTect SYBR Green PCR kit (Qiagen, Manchester, UK) was used to perform the amplification in duplicate, in the iCycleriQ Real-Time PCR Detection System (Applied Biosystem, Cheshire, UK), following the manufacturer’s protocols. Relative expression quantification was calculated using Rest 2009 version 2.0.13 software [31].

### Data analysis

Normally of distributions was checked by Shapiro-Wilk test and Kolmogorov-Smirnov and QQ plot. Data were compared between two groups using unpaired student’s t-test and the results were expressed as the mean ± SEM. Qualitative variables were expressed as percentages and *p*-values less than 0.05 were considered statistically significant. Statistical calculations were performed using GraphPad Prism Software Version 8.0 (GraphPad Software, Inc., USA).

## Results

**Fig. 1:**
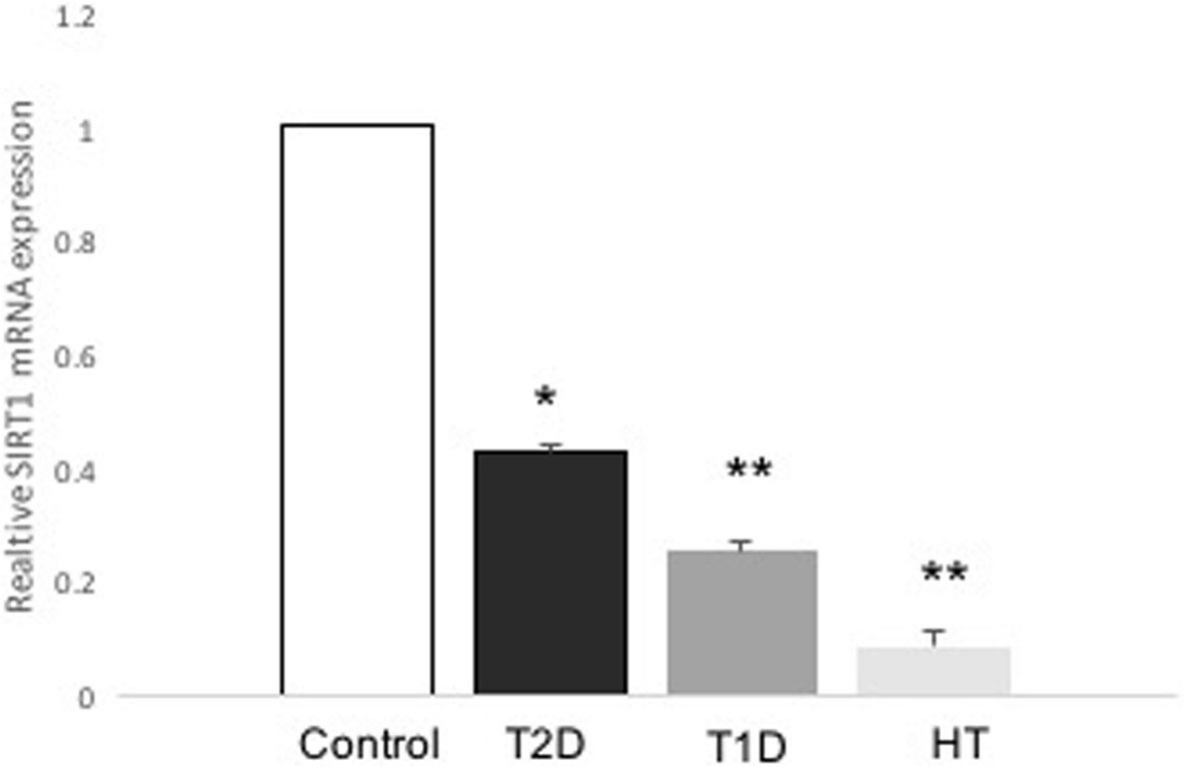
Expression levels of *SIRT1* in patients with T2D, T1D, HT and control individuals. The evaluation of the expression levels of *SIRT1* and *β-actin* are shown for control (*n* = 13), T2D (*n* = 22), T1D (*n* = 5), and HT (*n* = 7). *SIRT1* expression was measured by real-time PCR. Data are presented as means ± SEM. n donated the number of donors. *P < 0.05, **p < 0.01 versus *controls*

**Fig. 2:**
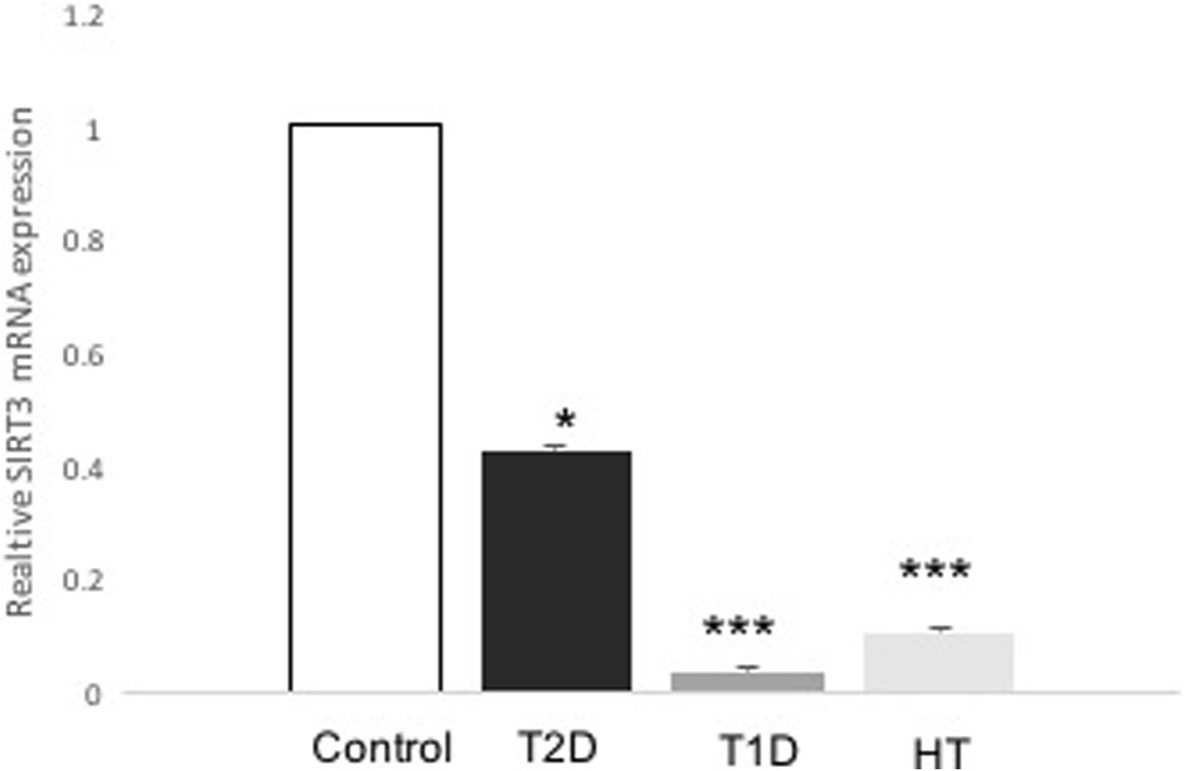
Expression levels of *SIRT3* in patients with T2D, T1D, HT and control individuals. The evaluation of the expression levels of *SIRT3* and *β-actin* are shown for control (*n* = 13), T2D (*n* = 22), T1D (*n* = 5), and HT (*n* = 7). *SIRT3* expression was measured by real-time PCR. Data are presented as means ± SEM. n donated the number of donors. *p < 0.05, ***p < 0.001 versus *controls*

**Fig. 3:**
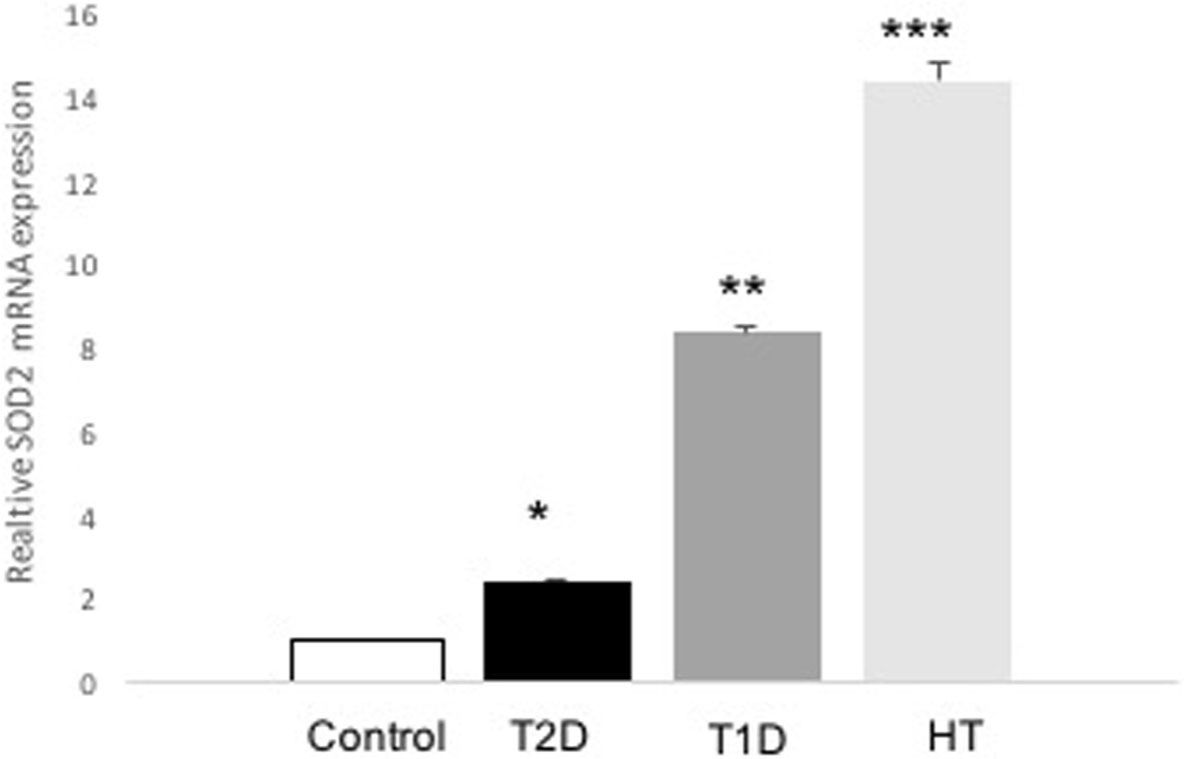
Expression levels of *SOD2* in patients with T2D, T1D, HT, and control individuals. The evaluation of the expression levels of *SOD2* and *β-actin* are shown for control (*n* = 13), T2D (*n* = 22), T1D (*n* = 5), and HT (*n* = 7). *SOD2* expression was measured by real-time PCR. Data are presented as means ± SEM. n donated the number of donors. *p < 0.05, **p < 0.01, ***p < 0.001 versus *controls*

**Fig. 4:**
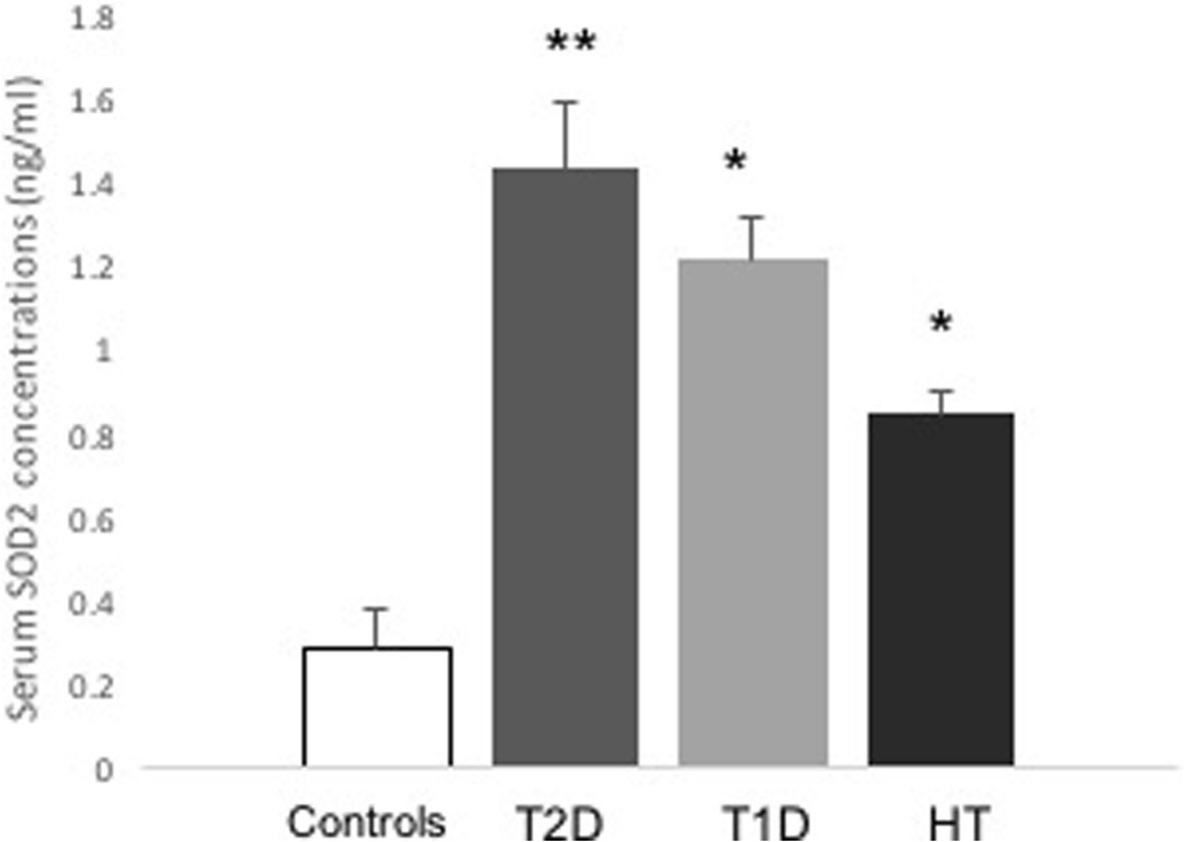
Serum concentrations of SOD2 in T2D, T1D, and HT. Secretion of SOD2 was measured by ELISA in control (*n* = 6), T2D (*n* = 4), T1D (*n* = 4) and HT (*n* = 4) (B). Data are presented as means ± SEM. n donated the number of donors*p < 0.05, **p < 0.01 versus *controls*

Fasting plasma glucose level was significantly higher in the T1D and T2D groups than in the healthy control group. The average age of the patients in the T2D group was also significantly higher than that in the healthy controls. There were no statistically significant differences in these above parameters between the HT and control group (Table 1). The serum level of TBARS was significantly higher in the HT group than in the healthy controls (*P* = 0.01, *n* = 3), as revealed in Table 1. Furthermore, there was not a statistically significant increase in the TBARS levels in the T2D group and no difference in those in the T1D group compared with the levels in the control group (Table 1). The expression of SIRT1 mRNA level was significantly lower (0.526-fold, *P* = 0.032, *n* = 13–22) in the patients with T2D than in the control group (Fig. 1). Similar levels were also observed in the patients with T1D (0.252-fold, *P* = 0.006 *n* = 5–13) and HT (0.385-fold, *P* = >0.008, *n* = 7–13) relative to those in the control group (Fig. 1). Interestingly, there was a significant decrease in the SIRT3 mRNA level in the T2D group compared with that in the control group (0.426-fold, *P* = 0.048, *n* = 13–22) (Fig. 2). The patients with T1D (0.027-fold, *P* < 0.001, *n* = 5–13) and HT (0.084-fold, *P* < 0.001, *n* = 7–13) also displayed a significantly lower level of SIRT3 mRNA than the control group (Fig. 2). As depicted in Fig. 3, there was a significantly higher level of *SOD2* in the T2D group than in the controls (2-fold, *P* = 0.011, *n* = 13–22). Similarly, we observed significantly higher levels of SOD2 mRNA in the T1D (8-fold, *P* = 0.001, *n* = 5–13) and HT groups (14-fold, *P* < 0.001, *n* = 7–13) than in the control group (Fig. 3). As expected, the SOD2 protein level was also significantly higher in the T2D group than in the control group (*P* = 0.0029, *n* = 4–6) (Fig. 4). Similar values were found in the T1D (*P* = 0.03, *n* = 4–6) and HT groups (*P* = 0.03, *n* = 4–6) relative to the control group values (Fig. 4).

**Table 1:** Clinical characteristics of subjects participating in the study

Status mothers	Controls (*n* = 13)	T2D (*n* = 22)	T1D (*n* = 5)	HT (*n* = 7)	*p* (T2D /T1D/HT) vs. controls)
Age (years)	45 ± 0.8	54 ± 0.3	29.2 ± 3	38 ± 1.5	**0.02**/0.12/0.3
Sex					
Female [n (%)]	6 (46)	12 (55)	2 (40)	7 (100)	
Male [n (%)]	7 (54)	10 (45)	3 (60)		
Duration (years)		12 ± 0.2	14 ± 0.1	11 ± 0.8	
Weight (kg)	82 ± 1	79 ± 0.5	86 ± 0.1	79 ± 2	0.07/0.4/0.9
BMI	29 ± 0.2	30 ± 0.3	30 ± 2	32 ± 0.8	0.5/0.4/0.4
HbA1c (%)	5.6 ± 0.02	8.2 ± 0.07	10 ± 0.2		**< 0.001**/< **0.001**
FPG (mM)	4.9 ± 0.04	10.9 ± 0.2	11 ± 0.3	4.7 ± 0.06	**< 0.001**/**< 0.001/**0.2
TSH (mIU/L)				9.12 ± 2	
Cholesterol (mmol/L)		4.2 ± 0.04		4.50 ± 0.1	
TBARS (nmol/mL)	7.83 ± 0.3 (*n* = 3)	8.67 ± 1 (*n* = 3)	7.46 ± 0.7 (*n* = 3)	15 ± 1 (*n* = 3)	0.3/0.4/**0.01**

Data are presented as mean ± SEM or n. *FPG* fasting plasma glucose, *BMI* Body mass index, *HbA1c* glycosylated hemoglobin, *TSH* Thyroid stimulating hormone. n denoted number of sample. Bold number indicated significant data

## Discussion

In this study, we found that the mRNA expression levels of *SIRT1* and *SIRT3* were decreased in patients with T2D, T1D, and HT, whereas the expression of SOD2 was increased at the mRNA and protein levels in all groups as compared with levels in the control group. These data could explain the genetic link between diabetes and HT.

Diabetes complications occur as a result of excessive free radical production, caused by damage to the antioxidative defenses or radical-induced enzyme inactivation [32]. Among the sirtuin family members, SIRT1 and SIRT3 proteins are required to reduce oxidative stress, as reported by previous studies [33–35]. In this study, *SIRT1* and *SIRT3* expression was decreased in T1D and T2D, suggested their roles in the pathogenesis of diabetes. Here, we could only confirm the protein expression of the upregulated genes, as the detection of protein from downregulated genes was below the limit of detection of the ELISA. Our results are consistent with those of a previous study that reported a decrease in SIRT1 activity in lymphocytes derived from patients with T2D [33]. In addition, our previous study showed that *SIRT3* mRNA expression was decreased in postpartum women with gestational diabetes mellitus and T2D [30]. The mechanisms behind the low expression of circulating *SIRT1* and *SIRT3* are not yet fully understood. Recently, it was reported that the expression levels of SIRT1 and SIRT3 mRNA and protein were decreased in the retinas of rats with early-stage diabetes; however, after injection treatment with an antioxidant glucagon-like peptide 1 analog, exendin-4 (EX4), the expression of both SIRT1 and SIRT3 was restored to normal levels [36]. In that same study, the expression levels of *SIRT1* and *SIRT3* were decreased and the ROS level was increased in a culture system of H_2_O_2_-treated R28 cells; however, with the addition of EX4, the levels of both sirtuins were increased and the ROS level was decreased [36]. It can be inferred from this present study that the observed decrease in *SIRT1* and *SIRT3* levels in our model may be, at least in part, due to oxidative stress, although we found a nonsignificant increase in lipid peroxidation in the T2D group and no difference in TBARS level between the T1D and control groups.

Interestingly, in this study, the mRNA and protein levels of SOD2, the principle antioxidative enzyme, were higher in both the T1D and T2D groups than in the control group. This result could be explained by a compensatory response to increasing mitochondrial ROS level owing to hyperglycemia in the patients with diabetes, as SOD2 is a mitochondrial free-radical-scavenging enzyme. Nevertheless, there are contradictory findings in the literature regarding the levels of SOD2 in patients with diabetes [37]. Our results are in line with a study conducted by Moussa, which reported increased SOD2 activity in patients with T1D and T2D [38]. However, our results contradicted studies that did not specify the SOD subtype and showed its activity to be lower in patients with T2D than in healthy controls [39, 40]. These conflicting data could be attributed to the differences in the patients’ ethnicity, nutrition, and environment, or the techniques used in each study.

On the other hand, thyroid dysfunction has been shown to be associated with oxidative stress [41–44]. With this perspective, proteins that protect against oxidative stress, such as SIRT1 and SIRT3, may be thought to be affected by low TH levels. With this perspective, proteins that protect against oxidative stress, such as SIRT1 and SIRT3, may be thought to be affected by low TH levels. Although a limited number of studies have shown the influence of TH (T3 and T4) on sirtuins, our results are in line with an animal study that used immunohistochemistry to reveal weaker staining of SIRT2 in ganglion cells derived from hypothyroid rat pups [45]. The authors of that study suggested that TH plays an important role in activating the sirtuin family of proteins, possibly through binding to their receptors. Moreover, it has been shown that reduced SIRT1 expression and activity may lead to diabetes in mice and humans [46, 47]. As diabetes mellitus is frequently associated with HT, these observed alterations could explain the link between diabetes and HT, based on the similar reduction of sirtuin levels in patients with T1D and T2D in the current study.

Furthermore, the levels of SOD2 in this study were increased at both the mRNA and protein levels in patients with HT relative to the control levels. This could be a compensatory mechanism in response to oxidative stress, as we noticed a higher level of TBARS in the patients with HT than in the control group, suggesting direct damage to the lipid structure by oxidative stress in the patient group. This is in line with a study that showed that the increase in the TBARS level in patients with HT indicated the presence of oxidative stress [48], which might be considered as a risk factor for cardiovascular disease development, similar to diabetes. As HT is often accompanied by diabetes, we selected non-diabetic patients with HT to rule out the effect of diabetes on oxidative stress.

It has been shown that elevated levels of TSH trigger cytokine expression, decrease antioxidant levels [49], and directly increase oxidative stress [50]. The variation in the levels of TH is the main cause of in vivo oxidative stress, owing to its important role in mitochondrial respiration. Reduced TH levels have been shown to induce oxidative stress and lipid peroxidation in the liver, heart, and smooth muscle [51], suggesting that HT could be a risk factor for increased oxidative stress, which could lead to other complications, such as diabetes. In the current study, although patients with HT were on thyroxine medication, the damaging effect of HT was only partially reversible with TH replacement therapy. A previous study showed that neurocognitive functioning in HT patients remained significantly defective despite thyroxine treatment, compared with that of euthyroid controls without HT [52]. Moreover, TH therapy has been shown to augment mitochondrial oxygen consumption and oxidative stress in patients with HT [53, 54]. In line with this, our study showed that oxidative stress is evident in patients with HT after the administration of thyroxine. Therefore, long-term antioxidant strategies, such as vitamin E therapy and an antioxidant diet, are advised along with the use of this drug, to protect against any complications that might develop.

This study has some limitations; for example, HbA1c values were not established for patients with HT, and TSH and cholesterol levels were not determined in all groups. In addition, there were no significant differences in characteristics among the study subjects, except for the significant increases in fasting glucose levels and HbA1c in the T1D and T2D groups, and a higher age in the T2D group. This suggested that the observed alterations in genes may be, at least, in part due to hyperglycemia.

## Conclusion

In conclusion, the changes in sirtuin expression, as observed in our study, may explain the failures of the diabetic and HT defense mechanisms against oxidative stress. The finding that SOD2 was increased at the mRNA and protein levels in all groups suggests its role in counteracting the observed oxidative stress in diabetes and HT. Thus, these alterations in the expression levels of sirtuins and superoxide dismutase in diabetes and HT may be related, at least in part, to the oxidative stress. Thus, designing drugs to enhance SIRT1 and SIRT3 expression and their activity would be useful to protect patients with diabetes and HT against the damaging effect of oxidative stress.

## Abbreviations

BMI: Body mass index
ELISA: Enzyme-linked immunosorbent assay
FPG: Fasting plasma glucose
HbA1c: Glycosylated hemoglobin: Hypothyroidism
SIRT: Sirtuin
SOD: Superoxide dismutase
T1D: Type 1 diabetes
T2D: Type 2 diabetes
TBARS: Thiobarbituric acid reactive substances
TH: Thyroid hormone
TSH: Thyroid stimulating hormone
